# A Rare Case of Paired Congenital Cervical Aneurysms in a Communicating Vein: Clinical and Imaging Findings in a Pediatric Patient

**DOI:** 10.3390/pathophysiology32020025

**Published:** 2025-06-06

**Authors:** Grigol Keshelava, Zurab Robakidze, Igor Mikadze

**Affiliations:** Department of Vascular Surgery, Clinic Healthycore, 0112 Tbilisi, Georgia; zurab.robakidze@yahoo.com (Z.R.); igor.mikadze@gmail.com (I.M.)

**Keywords:** venous aneurysm, communicating vein

## Abstract

A four-year-old female patient was admitted for evaluation after a mass on the right side of her neck was noticed during straining (Valsalva maneuver). The family first observed the mass when the patient was one year old, and noted that it gradually increased in size over time. A family history assessment revealed no known genetic disorders. The patient underwent neck ultrasonography and computed tomography angiography (CTA), which revealed two aneurysms in a right-sided communicating vein. One aneurysm was located above the jugular notch, and the other was located in the retro-parotid region. The presence of two venous aneurysms in a right-sided communicating vein—one above the jugular notch and the other in the retro-parotid region—suggests a rare and apparently benign congenital anomaly. The progressive enlargement of these malformations warrants close monitoring and surgical intervention, and long-term follow-up may be necessary to prevent complications such as thrombosis, rupture, or compression of adjacent structures.

## 1. Introduction

The conventional definition of a venous aneurysm describes it as the expansion of a venous segment to 1.5 times the diameter of the healthy segments located upstream and downstream of the affected area [1].

A venous aneurysm refers to an unusual enlargement of a vein and has been documented in various locations within the neck, with the internal and external jugular veins being the most common sites [2,3,4,5]. An aneurysm of the anterior jugular vein is even less common [6,7,8]. In this case report, we are discussing a communicating vein saccular aneurysm, which appears to be absent from the existing medical literature.

We present a case involving a 4-year-old girl who has a paired aneurysm of the communicating vein on the right side.

## 2. Case Description

**Figure 1:**
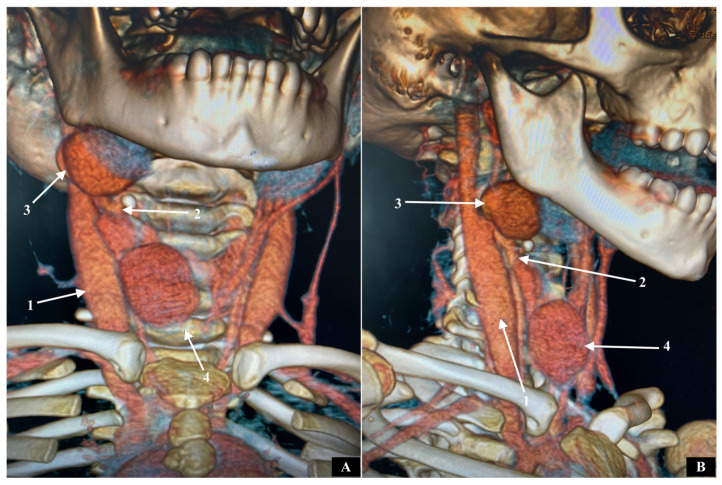
(**A**) Anterior view. (**B**) Right lateral view. CTA shows Two aneurysms of a right communicating vein. 1: right internal jugular vein; 2: right communicating vein; 3: aneurysm of the right communicating vein in the retro-parotid area; 4: aneurysm of the right communicating vein above the jugular notch.

The patient, a four-year-old female, was admitted to the clinic for a scheduled examination due to the presence of an asymptomatic tumor on the right side of the neck during tension (Valsalva maneuver). Family members first noticed this tumor one year after birth. It was also observed that it was increasing in size over time. The investigation of the parents did not reveal any genetic diseases. The patient underwent ultrasonography and computed tomography angiography (CTA) of the neck. Two saccular aneurysms of a right communicating vein were identified. CTA shows that one was located above the jugular notch (with maximum diameter 3.8 cm), while the other was on the retroparotid area (with maximum diameter 3.2 cm) (Figure 1A,B). This communicating vein was located along the anterior border of the sternocleidomastoid muscle and connected the anterior jugular vein to the internal jugular vein.

To prevent thrombosis, rupture and pulmonary thromboembolism [9,10,11], a surgical operation was offered–ligation of the venous branches from which blood was draining into the aneurysms, along with the excision of these aneurysms. The parents refused an operation. The patient was scheduled for follow-up ultrasonography once a year.

To the best of our knowledge no such case of paired congenital communicating vein aneurysm has been described in the medical literature.

## 3. Discussion

The origins of venous aneurysms are often unclear, but they are associated with conditions like nebulous hypertension and venous hypertension [12]. Additional potential causes include factors that weaken the venous wall, such as trauma, surgical procedures, infections, and genetic predispositions [7,13]. Based on their morphology, venous aneurysm, similar to arterial ones, can be classified as either saccular or fusiform.

Histologically, venous aneurysms are characterized by a thickened intima and a lack of smooth muscle layers, which may be deficient, or absent [14,15]. Furthermore, a recent study investigating the tissue of venous aneurysm indicated that localized structural changes might be connected to an increased expression of specific matrix metalloproteinases [16].

The differential diagnosis for this type of lesion comprises lymph nodes, laryngoceles, thyroid lesions, lipomas, thyroglossal cysts, cavernous hemangiomas, pharyngeal pouches, and arterial aneurysms [5]. The diagnostic methods utilized in the evaluation process encompass ultrasonography, venography, CT and MRI scans [12,17]. In our case ultrasonography and CT were conducted for diagnostic purposes.

The primary complications associated with venous aneurysms are thrombosis, thrombophlebitis, pulmonary thromboembolism and rupture [9,10,11].

Surgical removal is the preferred approach for managing venous aneurysms in the neck due to concerns about thrombosis, the potential for rupture, as well as cosmetic and aesthetic considerations [18]. Endovascular treatment or embolization offers a minimally invasive alternative that may yield comparable results and cosmetic advantages to surgery [19]. In the case we described, we believe it would have been more effective to remove the communicating vein along with both aneurysms, as this would have eliminated the risk of recurrence.

Understanding the anatomical variations in neck veins can be enhanced by examining key events during embryonic development. In 10 mm embryos, the primitive maxillary vein drains into the pericardial vein, while the ventral pharyngeal vein initially drains into the common cardinal vein and subsequently into the precardinal vein [20]. As development progress in 18 mm embryos, the ventral pharyngeal vein transforms into the linguofacial vein, into which the retromandibular vein begins to drain. At this stage, the primitive maxillary vein forms an anastomosis with the linguofacial vein. By the time the embryo reaches 40 mm, the external jugular vein connects to the retromandibular vein posteriorly and to the facial vein anteriorly. This anterior connection eventually degenerates before birth, leaving the facial vein solely connected to the internal jugular vein. The retromandibular vein remains linked to the internal jugular vein system through its anterior division and to the external jugular vein via its posterior division. Consequently, a persistent anterior connection of the primitive external jugular vein could result in different connections to the facial vein [20].

The Hamburg classification established in 1988 and modified in 1989 was introduced in an international consensus document by the International Society for the Study of Vascular Anomalies (ISSVA). This classification categorizes vascular anomalies into two primary groups: vascular tumors and hemangiomas. Each group is further divided into slow-flow and fast-flow categories, resulting in the identification of five types of malformations. These types are primarily arterial, predominantly venous, or primarily lymphatic, in addition to arteriovenous shunts and combined forms. Furthermore, these five categories are classified into truncular and extratruncular types [21]. In the case described above, it was identified as a truncular-type venous malformation.

Efforts to establish a universal classification for categorizing each case of this venous system are impractical because of the inherent individual variability. Each instance, particularly those requiring surgical intervention in the neck region, should be evaluated using medical imaging techniques such as MRI and CT scans for accurate assessment [22]. In the case we describe, the patient had two congenital aneurysms of the communicating vein, which connected the internal jugular vein to the anterior jugular vein.

## 4. Conclusions

This case highlights the importance of careful evaluation and monitoring in young patients presenting with neck masses. Given the potential for progressive enlargement and associated complications, ongoing surveillance and timely surgical intervention are critical. Moreover, imaging technic such as ultrasonography and CTA play a vital role in diagnosing and formulating effective treatment plans for vascular anomalies.

## Data Availability

No new data were created or analyzed in this study. Data sharing is not applicable to this article.
